# Citrus Peel Extracts for Industrial-Scale Production of Bio-Based Active Food Packaging

**DOI:** 10.3390/foods11010030

**Published:** 2021-12-23

**Authors:** Cecilia Fiorentini, Guillermo Duserm Garrido, Andrea Bassani, Claudia Cortimiglia, Marta Zaccone, Luana Montalbano, Vanesa Martinez-Nogues, Pier Sandro Cocconcelli, Giorgia Spigno

**Affiliations:** 1 Department for Sustainable Food Process (DiSTAS), Università Cattolica del Sacro Cuore, Via Emilia Parmense, 84, 29122 Piacenza, Italy; cecilia.fiorentini@unicatt.it (C.F.); guillermo.dusermgarrido@unicatt.it (G.D.G.); claudia.cortimiglia@unicatt.it (C.C.); pier.cocconcelli@unicatt.it (P.S.C.); giorgia.spigno@unicatt.it (G.S.); 2 Proplast, Via Roberto di Ferro 86, 15122 Alessandria, Italy; marta.zaccone@proplast.it (M.Z.); luana.montalbano@proplast.it (L.M.); 3 Tecnopackaging, Polígono Industrial Empresarium, Calle Romero 12, 50720 Zaragoza, Spain; vmartinez@tecnopackaging.com

**Keywords:** citrus peel extract, spray-drying, encapsulation, active food packaging, food compatibility

## Abstract

The thermal stability of four different commercial citrus peel extracts was tested and improved by an encapsulation process with β-cyclodextrins in a spray-dryer. All extracts after the encapsulation process maintained a good antioxidant capacity, with an apparent loss in total phenolic compounds of around 20–25%. In addition, all samples showed good antimicrobial activity (MIC 5–0.625 mg/mL) against *Staphylococcus aureus*, which was maintained after the encapsulation process (MIC 5–1.25 mg/mL). Based on the antioxidant and antimicrobial activity results, the best-encapsulated citrus extract was selected for incorporation into a polylactic acid/polyhydroxy butyrate (PLA/PHB) film. The latter was then produced on an industrial scale by cast extrusion and was found to be suitable for food contact as it showed overall migration values in different food simulants lower than the legislative limit of 10 mg of non-volatile substances per 1 dm^2^ of surface area. The UHPLC-HRMS analysis, performed to evaluate the migration of the active compounds, revealed about 13.41% release in food simulant A and 11.02% in food simulant B. Antimicrobial analysis conducted directly on the film showed a growth inhibition activity towards *Escherichia coli* and *Staphylococcus aureus* equal to 30 and 60%, respectively.

## 1. Introduction

Nowadays, the reduction of the potential risks associated with the use of synthetic compounds in food products and the limitations in using chemical additives are two hot-topics of research [1]. The incorporation of natural compounds in film or coating formulations to produce active packaging for food applications can be considered as a valid alternative [2]. Active packaging systems are designed to promote interactions between the packaging itself, the food, and the internal environment. In this framework, natural extracts represent a valuable source of bioactive compounds, such as polyphenols, characterized by well documented in vitro and in vivo antioxidant and antimicrobial properties [3,4] and they can be incorporated in or coated on food packaging materials to prevent microbial development, reduce lipid oxidation, and thereby extend the shelf-life of packaged foods [4,5,6]. Natural extracts can be obtained from different natural sources, such as fruits (e.g., grape and pomegranate), herbs and spices (e.g., tea, rosemary, oregano, and cinnamon), or, in a more sustainable view, from plant by-products, such as grapefruit seeds, grape pomace, orange peels, green tea and olive leaves, which are valued for their high functionality and/or bioactivity [1,7,8]. The latter is strictly dependent on the type of plant matrix, the extraction method, and the incorporation processes into the polymer [9,10].

The most widely used technique to include natural extracts into a film to produce active packaging is extrusion [11]. As a first step, this technique involves the incorporation of bioactive compounds in the matrix to be extruded to have an effective and homogeneous distribution of bioactive agents in the film. However, the high temperatures reached during the extrusion phase (typically around 180–200 °C) can lead to thermal degradation of the bioactive compounds, resulting in a decrease in their activity [12,13]. Therefore, to thermally protect the extracts, different microencapsulation techniques are often used like coacervation, molecular inclusion complexation, lyophilization, or spray-drying [14,15]. In particular, the spray-drying technique allows the formation of particles from a dispersion of a carrier agent (e.g., polysaccharides such as Arabic gum, maltodextrin, and cyclodextrins) in the liquid extract [16]. It is one of the most widely used microencapsulation methods in the food industry due to its low cost, simplicity of use, and easiness of scale-up compared to other drying processes such as lyophilization [17,18].

Starting from this background, the main goal of this study was to realize on an industrial scale an active film based on natural citrus extracts and PLA/PHB and to assess its active properties. Initially, four different commercial citrus peel extracts were characterized in terms of phenolic profile and in vitro bioactivity, and their thermal stability was improved through spray-drying encapsulation. Among these encapsulated extracts, the best was selected and tested for incorporation into an industrial scale PLA/PHB film. The latter was finally analyzed in terms of its activity and food contact compatibility.

## 2. Materials and Methods

### 2.1. Selected Commercial Citrus Extracts and Chemicals

Four different food-grade commercial citrus peel powder extracts were purchased from a Spanish company (Evesa, Cádiz, Spain). These extracts are different in the composition of bioactive compounds, such as the concentration of naringin, hesperidin, and neohesperidin. The kleptose/β-cyclodextrins (Roquette Services Techniques et Laboratories, France) were used as spray-drying carriers. The standards and reagents used were the following: gallic acid and hesperidin (Fluka, Buchs, Switzerland) Folin-Ciocalteu reagent (Fluka, Darmstadt, Germany), sodium carbonate (Na_2_CO_3_), ethanol (96% *v*/*v*), iron (III) chloride (FeCl_3_), 2,4,6-tripyridyl-s-triazine (TPTZ), 2,2’-azino-bis(3-ethylbenzothiazolin-6-sulfonate) (ABTS), 6-hydroxy-2,5,7,8-tetramethylchroman-2-carboxylic acid (Trolox) and hydrochloric acid (HCl) (Sigma-Aldrich, St. Louis, MO, USA). Luteolin (CAS number: 491-70-3), quercetin (CAS Number: 849061-97-8), and ferulic acid (CAS number: 1135-24-6) used as reference standards in the untargeted UHPLC-HRMS analysis were from Sigma-Aldrich (Milan, Italy).

### 2.2. Spray-Drying and Encapsulation Recovery Yields

The powder commercial citrus extracts were dissolved in NaOH (0.5% *w*/*v*) with a concentration of 10 mg/mL. To thermally protect the extracts through encapsulation, kleptose/β-cyclodextrins (β-CD) were added to the solution as carrier material with a dry ratio of 0.67 (*w*/*w*) powdered β-CD/powdered extract. The solution was then spray-dried in a mini spray drier (B-290, Buchi, Switzerland) at the following operation conditions fine-tuned in previous studies (data not shown): 4 mL/min feeding rate, aspirator rate set at 100% and 120 °C inlet air temperature. Finally, the spray-dried encapsulated extracts were collected and weighed immediately to calculate the recovery mass yield (%) with the following equation:

Recovery yield (%) = (g recovered powder on dry matter/g total solids of the feed on the dry matter) × 100.(1)

### 2.3. Characterization of Commercial Extracts and Spray-Dried Encapsulated Extracts

#### 2.3.1. Moisture Content and Water Activity

The dry matter content of the selected extracts before and after spray-dried encapsulation was evaluated according to the standard method [19]. Specifically, moisture content was calculated as weight loss of the samples (5 g) after 24 h in an oven at 105 °C.

A water activity meter (HygroPalm HP23-AW-A, Rotronic, Bassersdorf, Switzerland) was used to measure a_w_ at 25 °C of the selected powder extracts before and after encapsulation.

#### 2.3.2. Color Analysis

The color analysis of the extracts before and after encapsulation was performed with ColorFlex EZ (HunterLab) through the CIELab analysis, calibrated with a white standard tile. The results were expressed as color values of L*, a* and b*, where L* was used to denote lightness, a* redness, and greenness, and b* yellowness and blueness. The color values of each sample were measured in triplicate. The mean values were also processed to calculate chroma (C*) and the hue (H*) with the following formulas:

H* (°) = arctan (b*/a*) (2)

C* = (a*^2^ + b*^2^)^0.5^ (3)

High values of C* indicate brighter, more vivid colors, while H* (°) is a measure of color hue (H* = 0°, red; H* = 90°, yellow; H* = 180°, green; H* = 270°, blue).

#### 2.3.3. Differential Scanning Calorimetry

Thermal stability of powder extracts before and after encapsulation was assessed by differential scanning calorimetry (DSC) using DSC Setline (Setaram, Lyon, France) as reported by Shrestha et al. [20] with some modification. Aluminum closed crucibles with about 5 mg of samples were used for the test with a heating program from 30 to 300 °C at a heating rate of 5 °C/min.

#### 2.3.4. Particle Morphology

The particle morphology of all the encapsulated extracts was evaluated using a scanning electron microscope system (Fei Quanta FEG 250 Esem) in a high vacuum. The samples were processed with a mini deposition of Au (99.99% purity) by Sputtering Metal (Balzers MED 010) for high-resolution metallization.

#### 2.3.5. Total Phenolic Content and In Vitro Antioxidant Capacity (FRAP and ABTS Assays)

The total phenolic content (TPC) was analyzed using a microvolume version of the Folin-Ciocalteu assay [21]. The calibration curve was obtained with standard gallic acid in water (Fluka, 100–800 mg/L, *R^2^* = 0.999) and hesperidin in 0.1% NaOH solution (Fluka, 200–2000 mg/L, *R^2^* = 0.998). The results were expressed both as mg of gallic acid equivalents (GAE) and hesperidin equivalents (HE) on g of dry matter (dm) of extract.

Since natural extracts are composed of different phenolic compounds with different mechanisms of antioxidant activity, the antioxidant power of citrus extracts, before and after encapsulation, was measured using two antioxidant assays: the ferric reducing antioxidant power (FRAP) and ABTS assays.

The FRAP of each extract was evaluated according to the procedure described by Bassani et al. [21]. A calibration curve was prepared with FeSO_4_·7H_2_O in water (Carlo Erba, 0.2–2 mmolFe(II)/L, *R*^2^ = 0.999). Then the results were calculated and expressed as μmolFe(II)/g dm of extract.

The ability of the extract to reduce the ABTS radical was analyzed according to the method of Bassani et al. [21]. The calibration curve was obtained with a Trolox^®^ standard (Tr) in 50% ethanol (Sigma-Aldrich, 100–500 mgTr/L, *R*^2^ = 0.999) and the results were calculated and expressed as μmolTr/g dm of extract.

#### 2.3.6. Phenolic Profiling by Ultra-High-Pressure Liquid Chromatography (UHPLC) Coupled with High-Resolution Mass Spectrometry (HRMS)

The untargeted UHPLC-HRMS analysis was carried out to identify phenolic compounds present in the encapsulated citrus extract (ECE) and those migrated from the PLA/PHB+ECE film to food simulants A and B. The analysis was performed on a Q Exactive™_Focus Hybrid Quadrupole-Orbitrap Mass Spectrometer (Thermo Scientific, Waltham, MA, USA) coupled to a Vanquish ultra-high-pressure liquid chromatography (UHPLC) pump and equipped with heated electrospray ionization (HESI)-II probe (Thermo Scientific, USA), following the method reported by Rocchetti et al. [22], with some modifications. The chromatography was based on a mixture of water and acetonitrile (both LC-MS grade, from Sigma-Aldrich, Milan, Italy) as mobile phase both acidified with 0.1% formic acid, using an Agilent Zorbax Eclipse Plus C18 column (50 × 2.1 mm, 1.8 μm). The HRMS was based on a full scan analysis, under a positive ionization mode with a mass resolution of 70,000 at *m*/*z* 200. Pooled quality control (QC) samples were acquired in a data-dependent (Top *n* = 3) MS/MS mode to provide structural confirmations. The sequence of injections was randomized, and the injection volume was 6 µL. The HRMS data collection and elaboration were based on the software MS-DIAL (version 4.70), as previously detailed [22]. In particular, the identification step was based on mass accuracy, isotopic pattern, and spectral matching. The software MS-Finder was used for in-silico fragmentation of the not annotated mass features, using FoodDB and Phenol Explorer libraries, thus reaching a level 2 of confidence in annotation (i.e., putatively annotated compounds). The cumulative intensity values of the different phenolic classes annotated were then converted into semi-quantitative data, using methanolic solutions of pure standard compounds (Extrasynthese, Lyon, France) analyzed under the same conditions. Ferulic acid (phenolic acids), quercetin (flavonols), and luteolin (flavones and other flavonoids) were used as representatives of their respective classes. In this regard, a linear fitting (*R*^2^ > 0.99) was built and used for quantification.

Once the cumulative phenolics equivalent concentration for the encapsulated extract and the extract compounds in the food simulants had been assessed, the migration of the active compounds could be evaluated.

Based on the weight of the test specimen, the percentage of encapsulated extract in the film, and the volume of food simulant in contact with the film specimen, the maximum theoretical concentration of extract that could migrate into the simulant was calculated. Then, starting from this maximum theoretical extract concentration and the total phenolics equivalent concentration found with the UHPLC-HRMS analysis (injecting the encapsulated extract at a concentration of 10 ppm), the maximum theoretical concentration of total equivalent phenolics was calculated.

Finally, based on the ratio of the effectively migrated to the theoretical concentration of equivalent phenols for 100% migration, the % of migrated compounds was calculated.

#### 2.3.7. Antimicrobial Activity

The antimicrobial activity of all the extracts (before and after encapsulation) was assessed by microplate liquid dilution estimating the minimum inhibitory concentration (MIC), according to Wiegand et al. [23]. *Escherichia coli* ATCC 25922 and *Salmonella enterica* serovar Typhimurium DSM 17058 were tested using Mueller-Hinton broth (MHB) (Oxoid Ltd., Hampshire, UK), while *Listeria monocytogenes* DSM 15675 and *Staphylococcus aureus* ATCC 33591 were tested using cation-adjusted MHB (Oxoid Ltd., Hampshire, UK).

Briefly, fresh plate cultures were taken and re-suspended in a sterile saline solution until they reached final turbidity of 0.5 McFarland. The bacterial suspension was diluted 1:1000 times and used as inoculum.

The commercial natural extracts were tested at concentrations between 5 and 0.078 mg/mL, while the spray-dried encapsulated extracts were tested at concentrations between 10 and 0.156 mg/mL. To maintain sterility, all the extracts were filtered with 0.22 μm porosity filters (JET BIOFIL, Guangzhou, China).

In each well of the 96-well microtiter plate, 100 μL of double-concentrated culture medium, 50 μL of diluted extract, and 50 μL of bacterial inoculum were added. In addition, a positive control (without extract) and negative control (without bacteria inoculum) were added to each plate. A series of wells were also prepared using only the solvent in which the commercial natural extracts were solubilized (0.5% NaOH *w*/*v*) to verify that sodium hydroxide at this concentration did not inhibit the growth of the tested microorganisms. Samples were tested in triplicates and on different days. Finally, plates were incubated at 37 °C for 24 h; the growth was monitored by Multiskan Microplate Spectrophotometer (Themo Fisher Scientific) at a wavelength of 620 nm.

The results were expressed as MIC, i.e., the lowest concentration (mg/mL) capable of inhibiting the growth of the microorganism.

### 2.4. Film Production

A PLA/PHB film with the addition of the encapsulated citrus extract (labeled as PLA/PHB+ECE), was produced starting from the preparation of a homogeneous blend including PLA (Ingeo 2003D, NatureWorks, Minnetonka, MN, USA), PHB (produced in Newpack project from potato peels), a commercial liquid plasticizer (Glyplast^®^ OLA2, Condensia Quimica, Barcelona, Spain) and the encapsulated citrus extract (N40-60 E.) at a concentration minor than 2% wt. (the precise value cannot be reported due to Intellectual Property issues of the Newpack project). The blend was prepared using a co-rotating twin-screw extruder (Leistritz ZSE 27 MAXX, Nuremberg, Germany). The N40-60 E. extract was selected for this test because it showed good thermal stability, antimicrobial activity, both before and after encapsulation, towards all pathogenic microorganisms tested and antioxidant power comparable to the other tested citrus extracts.

The polymers and additives were dried to remove residual moisture at about 90 °C and 60 °C for 4 and 6 h, respectively. They were then fed through a gravimetric dosing unit (Brabender, Duisburg, Germany) at the beginning of the extrusion line through the main feeder, while the liquid plasticizer was fed at 60 °C via a peristaltic pump, positioned at two-thirds of the screw length from the extrusion die. The materials were extruded at approximately 170 °C with a screw speed of 250 rpm.

Then the cast extrusion process was used to produce the final film on an industrial scale, as it could not be processed by the air-blowing process (even after several changes in the process parameters). Specifically, the resulting blend was melted and extruded through a slot die onto a chilled/heated, highly polished, turning roll (chill roll) (Labtech, LCR-350). A PLA/PHB film produced without the addition of the encapsulated citrus extract was used as a control. The cast extruded sample had a final thickness of about 60–70 µm.

### 2.5. Overall Migration Test

The overall migration tests of the film were performed in triplicate in simulant A (10% (*v*/*v*) ethanol-water solution), simulant B (3% (*w*/*v*) acetic acid-water solution), and simulant D2 (olive oil) for 10 days at 40 °C in an oven, according to Regulation (EU) No 10/2011 [24]. For the experiment, specimens of the film of 1 dm^2^ were cut and folded to obtain a pouch in which one side of the film was in contact with the food simulant (reverse pouch). The pouches were then immersed in glass bottles containing 100 mL of each food simulant, according to European Standards EN-1186. Blank samples were run simultaneously in duplicate containing the simulant alone to check for contamination and make the blank correction in the evaluation of overall migration. The film without extract (PLA/PHB) was used as a control. The overall migration value (M) for both simulant A and B was expressed as mg of residue (non-volatile substances) per dm^2^ of the surface area of the sample intended to come into contact with food, and calculated with the following formula [25]:

M = (m_a_ − m_b_) × 1000/S (4)

where:

M: overall migration of the simulant,
m_a_: mass of the residue from the specimen (reverse pouch) after evaporation of the simulant in which it was immersed (g),
m_b_: mass of the simulant residue (blank) (g),
S: surface area of the pouch intended to come into contact with food (dm^2^).

While the overall migration value (M) for simulant D2 was expressed as mg of residue per dm^2^ of sample surface intended to come into contact with fatty food and calculated with the following formula [26]:

M = [m_a_ − (m_b_ − m_c_)] × 1000/S (5)

where:

M: overall migration in olive oil,
m_a_: initial mass of the specimen (reverse pouch) before contact with the simulant (g),
m_b_: mass of the specimen (reverse pouch) after contact with simulant (olive oil) (g),
m_c_: mass of oil absorbed by the specimen (reverse pouch) (g),
S: surface area of the specimen (reverse pouch) intended to come into contact with food (dm^2^).

### 2.6. Release of Bioactive Compounds

To assess whether the PLA/PHB+ECE film could be defined as active, tests were carried out to evaluate the release of bioactive compounds from the different samples to the food simulants used.

Both simulant A and simulant B, after contact for 10 days at 40 °C with the samples (reverse pouch), were recovered and tested directly for TPC (Folin-Ciocalteu assay), antioxidant capacity (FRAP assay), and the detection of phenolic compound subclasses migrated from the film (UHPLC-HMRS), following the procedures described above.

For the D2 simulant, oxitest analysis (Oxitest instrument, Velp-Scientifica, Varese, Italy) was performed directly on the simulant (olive oil) after contact with the PLA/PHB+ECE film to assess whether any release of antioxidants could slow down oxidation. In particular, the oxitest analysis was performed on 10 g of food simulant D2 after 0 (few seconds) and 10 days of contact with the PLA/PHB+ECE film at 40 °C, in the Oxitest set at 100 °C and initial pure oxygen pressure of 6 bar (operating conditions chosen based on previous tests not reported here). Oxygen consumption was monitored by recording the change in absolute pressure within the chambers of the instrument over time. The oxidation curve obtained was then processed by OXI Soft^TM^ software (Velp-Scientifica, Varese, Italy) to calculate the induction period (IP). A sample of olive oil kept in the same conditions as the sample, but not in contact with the film, was used as a control.

### 2.7. Antimicrobial Activity of the Film

For the obtained films, the antimicrobial activity was assessed according to the official method (ISO 22196:2011), to evaluate the possible antimicrobial effect of the citrus extract included in the PLA/PHB film (PLA/PHB+ECE) against *Escherichia coli* ATCC 8739 and *Staphylococcus aureus* ATCC 6538P.

Three specimens of 50 mm × 50 mm were cut from both PLA/PHB and PLA/PHB+ECE films. A bacterial suspension was prepared for each microorganism with a concentration of 2.5 × 10^6^ CFU/mL in a nutrient broth (NB) medium (Oxoid Ltd., Hampshire, UK). A specimen of the sample was placed in a Petri dish and 0.2 mL of the bacterial suspension was inoculated on its surface and subsequently, the inoculum was covered with a 40 mm × 40 mm commercial film. After inoculation, a count of viable bacteria recovered from the specimen was immediately carried out as a check on the viability of the inoculum. The plate was then incubated at 37 °C for 24 h. At the end of the incubation period, the inoculum was recovered by adding 10 mL of SCDLP broth (soybean casein digest broth with lecithin and polyoxyethylene sorbitan monooleate). Then, the count of viable cells was performed plating 1 mL of each 10-fold dilutions in Plate Count Agar (PCA) medium (Oxoid Ltd., Hampshire, UK) and incubating at 37 °C for 40–48 h. In the end, the number of viable bacteria recovered per mm^2^ was determined according to the following equation:

N = (C × D × V × 100)/S(6)

where:

N: number of viable bacteria recovered per cm^2^ per test specimen;
C: average plate count for the duplicate plates;
D: dilution factor for the plate counted;
V: volume of SCFLP added to the specimen (mL);
S: surface area of the covered film (mm^2^).

Finally, the average of the number of viable bacteria recovered from each sample was calculated and the antibacterial activity (R) value was determined, using the following formula:

R = [U_t_ − U_0_ − (A_t_ − U_0_)] = U_t_ − A_t_ (7)

where:

U_0_ = average of the common logarithm of the number of viable bacteria (cells/cm^2^), recovered from the untreated test specimens immediately after inoculum;
U_t_ = average of the common logarithm of the number of viable bacteria (cells/cm^2^), recovered from the untreated test specimens after 24 h;
A_t_ = average of the common logarithm of the number of viable bacteria (cells/cm^2^), recovered from the treated test specimens after 24 h.

### 2.8. Statistical Analysis

ANOVA analysis, with subsequent Tukey’s significant difference test (*p*-value of 0.05), was used to compare the data obtained from each experiment using IBM SPSS Statistics (Version 25; IBM, Armonk, NY, USA). All the data obtained in triplicate was reported as mean values ± standard deviation (SD).

## 3. Results and Discussion

### 3.1. Spray-Drying Encapsulation Yields

The yields (%) of the spray-drying encapsulation process resulted in more than 100% (Table 1) for all the tested extracts.

It is important to underline that the calculation of the final yield is probably overestimated since the extract was solubilized in 0.5% NaOH and sodium salts are probably formed, with this increasing the molecular weight of the compounds tested. This has been validated by trying to re-solubilize the encapsulated extract in water: they could be fully solubilized, although the pH of the final solution was higher than 10.

### 3.2. Characterization of Commercial Extracts and Spray-Dried Encapsulated Extracts

#### 3.2.1. Moisture Content, Water Activity, and Color

All the natural and the spray-dried encapsulated extracts were characterized in terms of moisture content, water activity, and color (Table 2).

The moisture content of each commercial natural extract was very low and significantly slightly increased after encapsulation. However, the overall moisture values remained very low (<5%), thus ensuring the stability of the obtained powder.

This stability was also confirmed by the low values of water activity, both before and after encapsulation.

The trichromatic coordinates of the commercial natural extracts, despite presenting some significant differences, appeared very similar to each other. However, the color values changed significantly after encapsulation with β-CD with particular reference to the trichromatic coordinate b* which represents the yellow color in positive values. In fact, after encapsulation, the extracts acquired a strong yellow color with different shades of tone. This was also confirmed by higher values of C* for encapsulated extracts that indicate more vivid and bright colors.

This further supports the hypothesis of the formation of sodium salts during the solubilization of the extracts in a sodium hydroxide (0.5% NaOH). Therefore, the formation of this color could represent a negative aspect for their incorporation in active food packaging depending on the addition level in the final film [6,27,28].

#### 3.2.2. Differential Scanning Calorimetry

Differential scanning calorimetry (DSC) was used to assess the thermal stability of the encapsulated natural extracts compared to the original ones and to confirm that encapsulation with β-CDs had occurred [29]. It is important to verify natural extracts’ stability at the material extrusion temperatures in order to include them in film for producing active packaging. Indeed, depending on the polymer and its melting temperature [12], high temperatures can be reached during extrusion (about 180–220 °C), which could lead to the degradation of the extract. Generally, as reported in several articles [13,17], pure natural extracts or additives degrade at that temperature.

**Figure 1:**
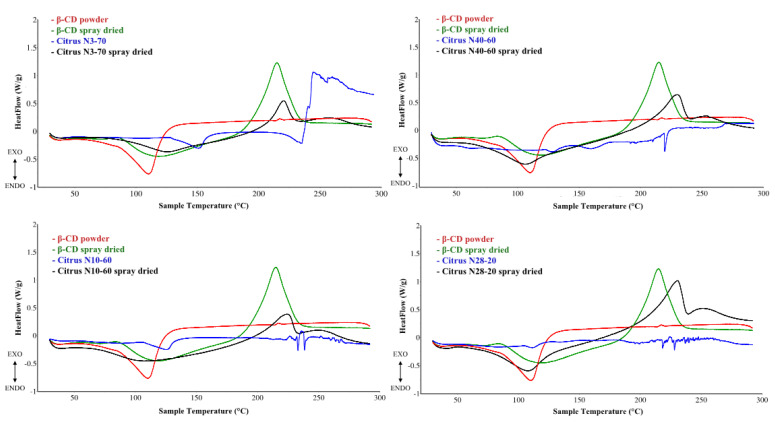
Comparison of thermal stability between commercial β-CD powder (red line), β-CD NaOH-solubilized and then sprayed (green line), started extracts (blue line) and encapsulated extracts (black line).

Figure 1 shows the thermal degradation profiles of natural extracts (blue line), encapsulated extracts (black line), β-CD used as a carrier (red line), and β-CD solubilized in a 0.5% NaOH solution (green line).

The DSC curve of β-CD showed the same profile as reported by Abarca et al. [30]. All the natural (not encapsulated) citrus extracts showed similar profiles (Figure 1).

An endothermic phenomenon can be observed between 120 and 150 °C, indicating evaporation of the residual moisture, while several exothermic and endothermic phenomena are generally observed between 180–250 °C, which could be due to thermal decomposition of the extracts [31,32]. For instance, Nascimento et al. [33] reported the DSC calorimetry curve of red propolis dry extract that showed peaks of simultaneous endothermic events in the range of 145 and 200 °C due to melting processes of the phenolic compounds. Moreover, as reported by Ficarra et al. [34], such endothermic phenomena could be related to the melt of hesperetin (226.73 °C), of hesperidin (263.19 °C), of naringenin (247.25 °C) and of naringin (249 °C). Therefore the DSC blue curves in Figure 1 could be associated with slightly different phenolic compositions of the citrus extracts.

After encapsulation with β-CD, the spray-dried extracts still showed an endothermic peak around 100 °C, as the one shown by β-CD due to both evaporation of residual moisture and a more homogeneous melting of the powder. This behavior was also observed by Nascimento et al. [33], that compared the calorimetry curves of microcapsules without red propolis dry extract and microcapsules with such extract. The curves showed similar trends, in which both exhibit endothermic events at the beginning, referring to water loss between 25 °C and 120 °C and, starting at 280 °C, an exothermic decomposition. Based on this, Nascimento et al. [33] state that there was microencapsulation of the extract and that there is also thermal compatibility.

In Figure 1 a single exothermic peak between 220–250 °C can be observed, which is not present in the β-CD powder. This peak could be due to the degradation of the sodium salts, that were probably formed after solubilization in NaOH, present in the encapsulated extract. Indeed, the same exothermic peak is also observable in a sample obtained from spray-drying of β-CD solubilized in a 0.5% NaOH solution (green line).

Since this unique exothermic phenomenon is observed for all four extracts at temperatures higher than extrusion ones (>200 °C), the encapsulated citrus extracts could be incorporated directly into polymer blends for film extrusion.

#### 3.2.3. Particle Morphology

**Figure 2:**
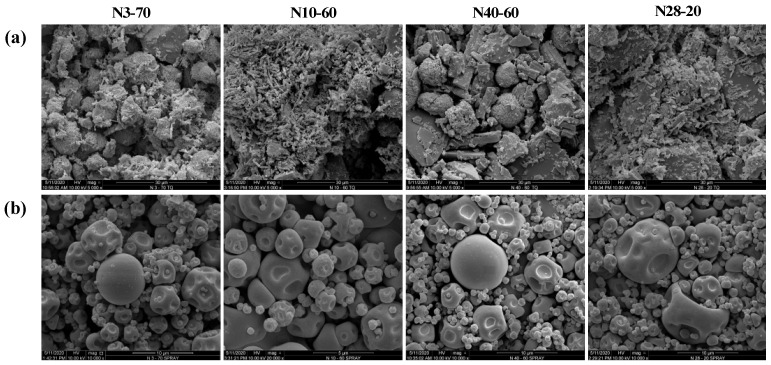
Observation of different citrus peel extracts before (**a**) and after (**b**) spray-drying encapsulation process with β-CD.

The scanning electron microscopy (SEM) analysis (Figure 2), was carried out to verify the actual change in the morphological characteristics due to the presence of the carrier material (β-CD), acting like a protective layer, following the spray-drying encapsulation process.

The SEM images show how the natural powder extracts are characterized by an amorphous and irregular shape, while after the spray-drying process with β-CD, the particles present a spherical shape and smooth surface, which is a typical feature of microparticles obtained by this encapsulation technique [35,36]. Although some particles present a “flat ball” effect, probably due to air bubbles originally generated during homogenization or atomization [16], no surface fractures are visibly confirming the successful encapsulation of citrus extracts with β-CD.

#### 3.2.4. Total Phenolic Content and In Vitro Antioxidant Capacity (FRAP and ABTS Assays)

After several solubility tests in distilled water and ethanol (data not shown), all extracts were found to be completely soluble in 0.5% NaOH at a maximum concentration of 20 mg/mL. On the other hand, the encapsulated extracts showed water solubility at a maximum concentration of 40 mg/mL. Therefore, the spray-drying encapsulation process was found to improve the water solubility of all the citrus extracts.

For this reason, to perform the liquid analysis, the natural and encapsulated extracts were solubilized at a concentration of 10 mg/mL in 0.5% NaOH (*w*/*v*) and distilled water, respectively.

The total phenolic contents are reported in Table 3 and, for the sake of comparison, are expressed both as mgGAE/g dm and as mgHE/g dm.

Citrus samples showed similar values of TPC expressed as mgHE/g dm, because hesperidin is usually the most representative phenolic compound of citrus fruits [37], even though the different extracts were selected to cover different percentages of hesperidin, naringenin, and neohesperidin. Overall, the extract with the highest content of polyphenols resulted in N3-70.

The TPC of the encapsulated extracts is expressed both as mgGAE/g_dm_ and as mgGAE/g of extracts (g_EXT_). As expected, the concentration of polyphenols, expressed as mgGAE/g dm, was less than the natural extract, likely due to the presence of β-CD in the powder obtained at the end of the spray-drying process. However, assuming the ratios between the extract and the cyclodextrins remained constant during the process and the weight of β-CD is not considered, the values (expressed as mgGAE/g_EXT_ dm) slightly increased, with an apparent loss in phenolic compounds (around 25%) for all the tested citrus extracts. These values are in line with those reported in the study by Shofinita et al. [38] in which several citrus extracts were encapsulated with whey protein isolate using the spray-drying process (with drying air inlet temperatures of 125 °C). At the end of the process, Shofinita et al. [38] obtained average TPC recovery values of around 80% for all the tested citrus extracts.

The % loss in phenolic compounds could be due to the air inlet temperature used. As reported by Ramírez et al. [39], when temperatures above 105 °C are used in the spray-drying process, the resulting excessive evaporation could cause cracks and deformation in the wall materials, leading to premature release of their contents and degradation of the encapsulated ingredient.

**Figure 3:**
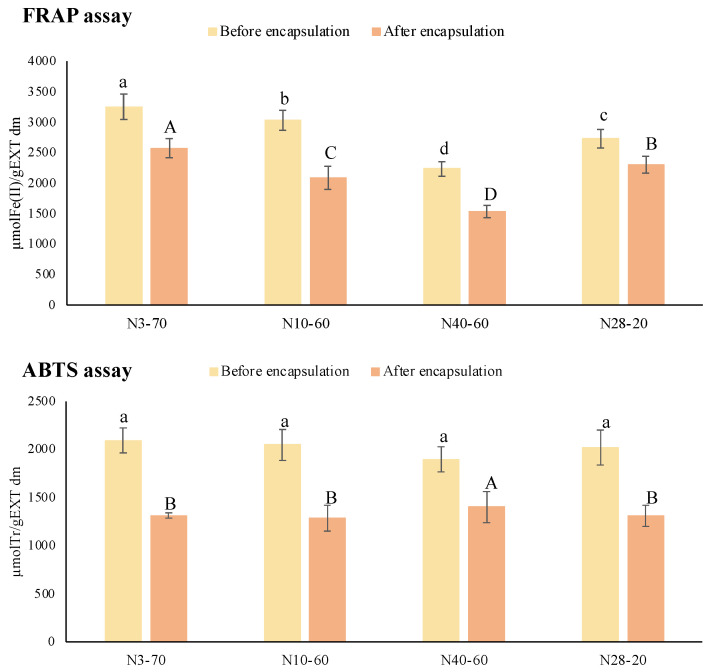
In vitro antioxidant capacity (evaluated with FRAP and ABTS assays) of extracts before (yellow) and after spray-drying (orange) with β-CD. Values reported on extract (EXT) dry matter (dm). Tr: Trolox. Error bars indicate ± standard deviation of mean values. Lowercase letters indicate comparisons between initial commercial extracts, while uppercase letters indicate comparisons between the encapsulated ones.

Since natural extracts are composed of different phenolic compounds with different mechanisms of antioxidant capacity, the in vitro antioxidant power of citrus extracts, before and after encapsulation, was measured using the FRAP and ABTS assays, evaluating the ability of the tested extracts to reduce iron and ABTS radicals, respectively (Figure 3).

As for the TPC, even the antioxidant capacity after the encapsulation process appeared to be slightly reduced, and in line with the TPC, the N3-70 commercial natural extract revealed the highest reducing capacity both for FRAP and ABTS assays. Moreover, among the encapsulated citrus extracts, extract N3-70 was confirmed to have one of the highest antioxidant activities.

#### 3.2.5. Antimicrobial Activity

The antimicrobial activity, expressed as the Minimum Inhibitory Concentration (MIC), of the commercial and encapsulated extracts was evaluated against the most common pathogens growing on food products, taking into consideration both Gram-positive and Gram-negative microorganisms. As reported in Table 4, all commercial citrus extracts showed good antimicrobial activity (MIC 5-0.625 mg/mL) against Staphylococcus aureus, in line with data reported on the literature [40,41]. Results showed that all extracts are potentially more active against S. aureus with respect to the other bacteria since the MIC values were lower. Concerning the other microorganisms (*E. coli, L. monocytogenes,* and *S. enterica*), it was not possible to determine the N3-70 MIC value, because the limited solubility of the extracts led to the use of a maximum concentration of 5 mg/mL, which was not effective. Despite this, the encapsulation process allowed to obtain an effective inhibitory concentration against *L. monocytogenes*. A similar situation was observed for the other extracts. Again, the antimicrobial activity of N10-60 was not determined for the commercial extract, but it was measurable after encapsulation. As demonstrated by other authors [42,43], the encapsulation procedure increased the efficiency of some extracts, considering the 0.67 ratio (*w*/*w*) between β-CD and extract. This behavior could be due to the increasing stabilization and solubilization of bioactive compounds [44]. These results were partially confirmed for all the extracts tested against S. aureus. Indeed, the MIC values of encapsulated N40-60 and N28-20 were 4 times increased with respect to natural extracts. This evidence led to suppose that the efficiency depends on the strain, as previously speculated [44].

Potentially, all these citrus extracts could be used as additives for the production of active food packaging useful for extending food shelf-life [45]. However, among the tested extracts, the one showing a lower MIC value against all the microorganisms was citrus extract N40-60. For this reason, it was selected to be added in the formulation of an active PLA/PHB-based film produced on an industrial scale.

### 3.3. Overall Migration Test

The overall migration test with food simulants, in accordance with Regulation (EU) No 10/2011 [24], allowed to evaluate the total mass of non-volatile substances that could migrate from the film to the food product, simulated using the food simulants A (10% ethanol *v*/*v*), B (3% acetic acid *w*/*v*), and D2 (olive oil).

As reported in Table 5, both the films tested showed overall migration values considerably lower than the legislative limit of 10 mg/dm^2^, making them potentially suitable for food contact.

Among the three food simulants tested, the statistically highest overall migration value was observed, for both films, in simulant D2. However, it must be considered that Regulation (EU) No 10/2011 [24] indicates that for some fatty food categories the result of the migration test with this food simulant must be divided by a certain value (between 2 and 5) before comparing the result with the migration limit. Therefore, the migration values obtained in simulant D2 could be even lower.

### 3.4. Release of Bioactive Compounds

To assess whether the PLA/PHB+ECE film could be defined as “active”, additional tests were performed to evaluate the release of active compounds from the samples to the food simulant used.

**Figure 4:**
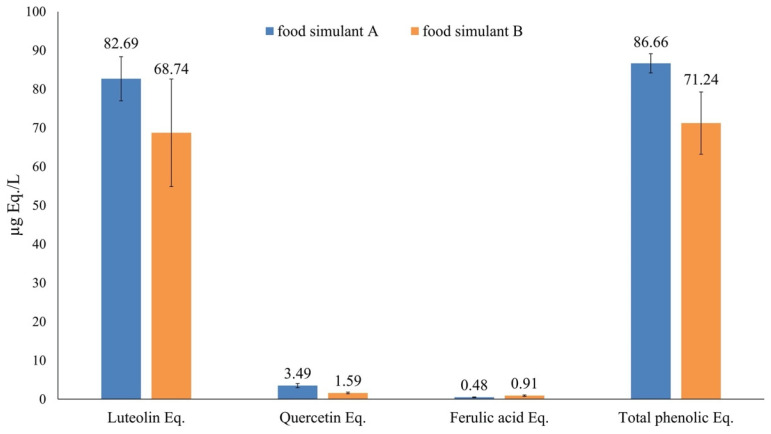
Evaluation of total phenolic equivalent concentration for the food simulants A (blue) and B (orange) after the migration of active compounds from PLA/PHB+ECE films. Error bars indicate ± standard deviation of mean values.

Therefore, the total phenolic compounds released from the PLA/PHB+ECE film into the different simulants after 10 days of contact at 40 °C were evaluated using the Folin-Ciocalteu assay. However, the absorbance values were not detectable. For this reason, the untargeted UHPLC-HRMS analysis was performed to better investigate a possible release of active compounds from PLA/PHB+ECE film to the food simulant used, and the results obtained are reported in Figure 4. The concentration of total phenolic compounds was particularly important to assess the real migration of active compounds, and it was evaluated as the sum of the relative abundance of the different compounds identified by the untargeted UHPLC-HRMS analysis and then expressed as equivalents of the standard compounds used.

The untargeted phenolic profiling approach allowed the putative identification of 16 compounds, being mainly composed of flavonoids, such as flavones, flavanones, and isoflavonoids. A comprehensive list reporting each compound together with its class, isotopic MS profile, and raw abundance values is reported in Table S1. The UHPLC-HRMS approach revealed alkaline hydrolysis of hesperidin (i.e., the most abundant compound) to produce its aglycone, namely hesperetin. This trend was in accordance with the information reported in the literature [46], stating that hesperidin may undergo alkaline hydrolysis at high pH, among the other potential conditions. Besides, the profiling approach revealed other typical citrus phenolics [37], such as naringin, neohesperidin, chalconaringenin, and other glycosylated flavones (Table S1).

As reported in the materials and methods section, once the total phenolics equivalent concentration for both the encapsulated extract and each migrated extract in the food simulants had been assessed, the migration of the active compounds was evaluated.

Based on the weight of the test specimen (average weight of 0.5145 g), the percentage of encapsulated extract present in the film (<2% wt.) and the volume of food simulant (100 mL) in contact with the film, the maximum theoretical concentration of extract that could migrate into the simulant was evaluated. Then, starting from this maximum theoretical extract concentration (corresponding to 100% migration) and the total phenolics equivalent concentration found with the UHPLC-HRMS analysis (125.59 µg Eq./L) (injecting the encapsulated extract at a concentration of 10 ppm), the maximum theoretical concentration of total equivalent phenolics was found to be 646.16 ± 98.43 µg/L.

As shown in Figure 4, the found concentration of total equivalent phenols was lower, for both food simulants, than the calculated theoretical maximum value. This could be due to a strong bonding effect between film and extract generated during compounding and to thermal degradation of the active compounds during cast extrusion, as well.

A slightly higher % of migration was observed in simulant A than in simulant B (13.41 ± 0.47% and 11.02 ± 1.52%, respectively), but without significant statistical differences.

**Figure 5:**
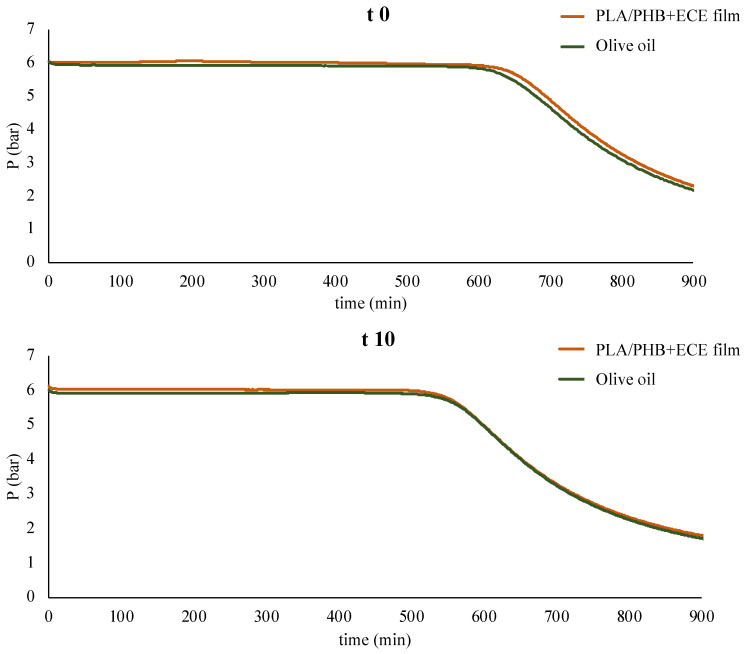
Oxitest analysis after 0 (t 0) and 10 (t 10) days of contact between the PLA/PHB+ECE film (orange) and the food simulant D2 (green).

The samples tested with the food simulant D2 were analyzed with the oxitest assay to assess whether any release of antioxidants from the PLA/PHB+ECE film could slow down oxidation. In this analysis, the stability of the sample is determined by accelerating the oxidation process through the combined effect of high temperature and pressure. The analysis gives the induction period (IP), corresponding to the time value at which the pressure line starts descending (Figure 5). The longer the IP, the higher the stability against oxidation.

However, both at time 0 (few seconds) and after 10 days, the PLA/PHB+ECE sample showed a similar trend to the control sample (Figure 5). Therefore, any substance released from the film to the food simulant did not seem to improve the oxidation resistance of the olive oil.

Even if the % of migrated substances results much lower than 100%, in future studies, the film containing the encapsulated citrus extract could be tested to be applied in contact with food (e.g., fruit, vegetables, or processed meat products) to verify a possible extension of their shelf-life. Indeed, the migration rate also depends on other factors such as peculiar food characteristics (composition, pH, and a_w_) and the relative humidity of the storage condition that, with food simulants, are not fully considered [2,47]. That’s why it will be important to test the film in real food contact tests.

### 3.5. Antimicrobial Activity of the Film

Since the encapsulated N40-60 citrus extract was demonstrated to have the lowest MIC values against the selected spoiling microorganisms, this extract was selected to further inclusion in a PLA/PHB film. Then the antimicrobial activity of the film was evaluated as a percentage of inhibition in the microorganism’s growth by comparing the treated and untreated samples. The results showed a percentage of inhibition to E. coli equal to 30.06% (R-value 0.16), while inhibition of 61.15% to S. aureus (R-value 0.41). This demonstrated that the PLA/PHB+ECE film did not completely inhibit the growth of none of the tested microorganisms. However, it was more effective against S. aureus with a percentage of microbial growth inhibition twice that of E. coli, confirming the inhibitory efficacy detected in the MIC assay.

Therefore, the produced PLA/PHB+ECE film, if tested in contact with food, may not completely inhibit the microbial growth but at least slow it down. This could allow the extension of the shelf-life of a food product by a few days that, anyway, might be an important achievement for products with very short shelf lives.

## 4. Conclusions

The thermal stability of four different commercial citrus peel extracts was improved by encapsulation with β-cyclodextrins (β-CD) in a spray-dryer. The encapsulation process did not greatly reduce the antioxidant capacity of the extracts, with an apparent loss of total phenolic compounds around 20–25%. In addition, all samples showed good antimicrobial activity (MIC 5–0.625 mg/mL) against *Staphylococcus aureus*, which was maintained even after encapsulation (MIC 5–1.25 mg/mL).

The PLA/PHB film produced on an industrial scale with the addition of one selected encapsulated citrus extract, was found to be potentially suitable for food contact as it showed overall migration values significantly below the legislative limit of 10 mg/dm^2^ for the tested food simulants (A, B and D2). The UHPLC-HRMS analysis, performed to evaluate the migration of active compounds, showed a release of about 13% in food simulant A and 11% in food simulant B. Antimicrobial analysis conducted directly on the film showed growth inhibition activity towards *Escherichia coli* and *Staphylococcus aureus* of 30 and 60%, respectively.

Future studies should focus on testing the innovative produced film directly in contact with fresh foods in order to evaluate a possible increase in their shelf-life.

## Figures and Tables

**Table 1:** Recovery mass yields (% on dry matter, dm) of the spray-drying encapsulation process. Values reported as mean ± standard deviation. Values reported in different lowercase letters are significantly different (*p* < 0.05).

Encapsulated Extracts	Recovery Mass Yields (% dm)
N3-70	119.25 ± 1.36 ^a^
N10-60	104.17 ± 1.19 ^c^
N40-60	120.95 ± 1.27 ^a^
N28-20	109.11 ± 1.70 ^b^

**Table 2:** The moisture, water activity (a_w_), trichromatic coordinates (L*, a*, and b*), H* (°) and C* values of the natural extracts and spray-dried encapsulated (E.) extracts. Values reported as mean ± standard deviation. Lowercase letters indicate comparisons between initial commercial extracts, while uppercase letters indicate comparisons between the encapsulated ones.

Citrus Extracts before and after Encapsulation	Moisture (%)	Water Activity (a_w_)	Trichromatic Coordinates			H* (°)	C*	Color
			L*	a*	b*			
**N3-70**	2.67 ± 0.02 ^b^	0.26 ± 0.01 ^c^	74.80 ± 0.89 ^a^	2.18 ± 0.29 ^c^	20.19 ± 1.40 ^a^	83.84	20.31	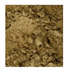
**N3-70 E.**	4.84 ± 0.30 ^CB^	0.29 ± 0.01 ^A^	66.94 ± 0.01 ^A^	15.71 ± 0.01 ^D^	74.02 ± 0.02 ^B^	78.02	75.67	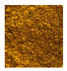
**N10-60**	1.18 ± 0.02 ^d^	0.27 ± 0.01 ^b^	72.80 ± 0.50 ^b^	3.93 ± 0.33 ^b^	16.64 ± 1.96 ^b^	76.71	17.10	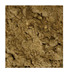
**N10-60 E.**	4.60 ± 0.02 ^C^	0.21 ± 0.01 ^B^	63.66 ± 0.01 ^B^	16.95 ± 0.01 ^C^	71.70 ± 0.10 ^C^	76.70	73.68	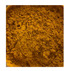
**N40-60**	4.19 ± 0.02 ^a^	0.40 ± 0.01 ^a^	70.86 ± 0.19 ^c^	4.08 ± 0.22 ^b^	17.97 ± 0.59 ^ab^	77.21	18.43	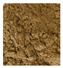
**N40-60 E.**	5.38 ± 0.02 ^A^	0.21 ± 0.01 ^B^	61.95 ± 0.01 ^C^	19.89 ± 0.01 ^A^	75.99 ± 0.03 ^A^	75.33	78.55	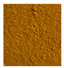
**N28-20**	2.02 ± 0.03 ^c^	0.40 ± 0.01 ^a^	71.27 ± 0.12 ^c^	5.07 ± 0.10 ^a^	18.59 ± 0.19 ^ab^	74.74	19.27	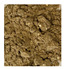
**N28-20 E.**	5.22 ± 0.05 ^AB^	0.21 ± 0.01 ^B^	60.74 ± 0.01 ^D^	17.55 ± 0.01 ^B^	70.00 ± 0.01 ^D^	75.93	72.17	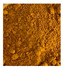

**Table 3:** Total phenolic content (GAE gallic acid equivalents, HE hesperidin equivalents) of the citrus extract before and after encapsulation with β-CD. Values reported on powder (g) or on extract (EXT) dry matter (dm) as mean ± standard deviation. Lowercase letters indicate comparisons between initial commercial extracts, while uppercase letters indicate comparisons between the encapsulated ones.

CITRUS EXTRACT	BEFORE Encapsulation		AFTER Encapsulation			
	mgGAE/g dm	mgHE/g dm	mgGAE/g dm	mgGAE/g_EXT_ dm	mgHE/g dm	mgHE/g_EXT_ dm
**N3-70**	315.09 ± 19.44 ^a^	963.13 ± 82.89 ^a^	142.19 ± 8.35 ^A^	235.68 ± 13.84 ^A^	422.28 ± 37.29 ^A^	699.94 ± 61.80 ^A^
**N10-60**	286.16 ± 17.54 ^b^	858.63 ± 72.10 ^b^	130.38 ± 7.78 ^A^	217.74 ± 12.99 ^A^	379.23 ± 34.99 ^AB^	633.31 ± 58.44 ^A^
**N40-60**	274.36 ± 16.11 ^b^	809.71 ± 64.08 ^b^	116.19 ± 11.27 ^B^	194.03 ± 18.82 ^B^	329.60 ± 53.26 ^B^	550.38 ± 88.93 ^B^
**N28-20**	282.19 ± 17.87 ^b^	844.29 ± 60.93 ^b^	130.30 ± 10.37 ^A^	217.18 ± 17.29 ^A^	378.66 ± 24.70 ^AB^	631.11 ± 41.16 ^AB^

**Table 4:** Antimicrobial activity of commercial and encapsulated natural extracts expressed as minimum inhibitory concentration (MIC). Each value is reported as the mean of three replicates.

Citrus Extracts before and after Encapsulation	*Listeria* *monocytogenes* DSM 15675	*Escherichia coli* ATCC 25922	*Salmonella* *enterica* DSM 17058	*Staphylococcus* *aureus* ATCC 33591
	MIC mg/mL			
**N3-70**	>5	>5	>5	1.25
**N3-70 E.**	10	>10	>10	2.5
**N10-60**	>5	>5	>5	1.25
**N10-60 E.**	10	10	10	2.5
**N40-60**	5	5	5	0.625
**N40-60 E.**	10	10	10	2.5
**N28-20**	>5	5	5	0.625
**N28-20 E.**	10	10	10	2.5

**Table 5:** Overall migration values obtained for the tested films in contact with food simulants A, B, and D2. Values expressed as mean ± standard deviation. Values reported different lowercase letters are significantly different (*p* < 0.05).

Film	M (mg/dm^2^)		
	Simulant A	Simulant B	Simulant D2
**PLA/PHB**	1.07 ± 0.20 ^a^	1.14 ± 0.32 ^a^	6.00 ± 1.11 ^b^
**PLA/PHB+ECE**	1.00 ± 0.13 ^a^	0.56 ± 0.21 ^a^	7.45 ± 1.12 ^b^

## Data Availability

Not applicable.

## Supplementary Materials

The following are available online at https://www.mdpi.com/article/10.3390/foods11010030/s1, Table S1: phenolic profiling dataset resulting from the UHPLC-HRMS annotation workflow.
